# Sulfatide imaging identifies tumor cells in colorectal cancer peritoneal metastases

**DOI:** 10.1186/s40170-024-00345-3

**Published:** 2024-06-28

**Authors:** G. M. Sarcinelli, L. Varinelli, S. Ghislanzoni, F. Padelli, D. Lorenzini, A. Vingiani, M. Milione, M. Guaglio, S. Kusamura, M. Deraco, G. Pruneri, M. Gariboldi, D. Baratti, I. Bongarzone

**Affiliations:** 1 Department of Diagnostic Innovation, Fondazione IRCCS Istituto Nazionale Dei Tumori, Via G. Amadeo 42, 20133 Milan, Italy; 2 Department of Research, Fondazione IRCCS Istituto Nazionale Dei Tumori, Via G. Amadeo 42, 20133 Milan, Italy; 3 Department of Pathology and Laboratory Medicine, Fondazione IRCCS Istituto Nazionale Dei Tumori, Via G. Venezian 1, 20133 Milan, Italy; 4 Department of Diagnostic Innovation, Fondazione IRCCS Istituto Nazionale Dei Tumori, Via G. Venezian 1, 20133 Milan, Italy; 5 Peritoneal Surface Malignancies Unit, Fondazione IRCCS Istituto Nazionale Dei Tumori, Via G. Venezian 1, 20133 Milan, Italy

**Keywords:** Peritoneal carcinomatosis, Lipid, Sulfatide, MALDI-imaging, Organoids

## Abstract

Even with systemic chemotherapy, cytoreductive surgery (CRS), and hyperthermic intraperitoneal chemotherapy (HIPEC), peritoneal metastases (PM) remain a common site of disease progression for colorectal cancer (CRC) and are frequently associated with a poor prognosis. The mass spectrometry (MS) method known as Matrix-Assisted Laser Desorption/Ionization – Time of Flight (MALDI-TOF) is frequently used in medicine to identify structural compounds and biomarkers. It has been demonstrated that lipids are crucial in mediating the aggressive growth of tumors. In order to investigate the lipid profiles, particularly with regard to histological distribution, we used MALDI-TOF MS (MALDI-MS) and MALDI-TOF imaging MS (MALDI-IMS) on patient-derived tumor organoids (PDOs) and PM clinical samples. According to the MALDI-IMS research shown here, the predominant lipid signature of PDOs in PM tissues, glycosphingolipid (GSL) sulfates or sulfatides, or STs, is unique to the areas containing tumor cells and absent from the surrounding stromal compartments. Bioactive lipids are derived from arachidonic acid (AA), and AA-containing phosphatidylinositol (PI), or PI (18:0–20:4), is shown to be highly expressed in the stromal components. On the other hand, the tumor components contained a higher abundance of PI species with shorter and more saturated acyl chains (C34 and C36 carbons). The cellular subversion of PI and ST species may alter in ways that promote the growth, aggressiveness, and metastasis of tumor cells. Together, these findings suggest that the GSL/ST metabolic programming of PM may contain novel therapeutic targets to impede or halt PM progression.

**Supplementary Information:**

The online version contains supplementary material available at 10.1186/s40170-024-00345-3.

## Introduction

Colorectal cancer (CRC) is the third most common tumor and the second cause of cancer-related death worldwide [51]. Peritoneal metastases (PM) from CRC, are a primary cause of patient morbidity and mortality, as in recent population-based studies the prevalence of synchronous PM was 3.8–5.7%, second only to liver metastases, and the incidence of metachronous disease was 3.5–5.9% [22, 28, 46, 58]. PM are less responsive to systemic chemotherapy and associated with a poorer prognosis, as compared with extra-peritoneal metastatic sites like the liver and lung [9]. Historically, CRC-PM have always been considered an end-stage disseminated disease, only to be treated with palliative care. However, a better understanding of the patterns of disease progression has made it clear that the peritoneum represents the only site of metastatic disease in 41–45% of patients. Accordingly, PM are currently seen as a locoregional disease stage, and a growing number of patients are treated by cytoreductive surgery (CRS) coupled with hyperthermic intraperitoneal chemotherapy (HIPEC).

In recent decades, the prognosis of CRC-PM treated by CRS/HIPEC has improved and long-term survival is now possible [1, 54, 58]. However, only highly selected patients can benefit from CRS/HIPEC, and the majority of them is excluded from combined treatment because of advanced peritoneal involvement, poor clinical conditions, and/or systemic metastases. Additionally, more than 50–60% of patients undergoing CRS-HIPEC experience disease recurrence even after optimal treatments [1].

It is still unclear what mechanisms control the PM's capacity to spread and why the prognosis for PM is so bad. Currently, a number of molecular classifications for CRC have been proposed. These include the presence or absence of driver mutations (such as those in the RAS and BRAF genes), the stability or instability of microsatellite instability, the consensus molecular subtype (CMS), and the intrinsic subtypes of CRC (CRIS) [43]. The relationship between these molecular characteristics and the organotropism of peritoneal metastases derived from colorectal cancer is, however, poorly understood [37].

Lipid metabolism has gained increased attention in cancer research recently [35]. It is widely known that CRC cells exhibit aberrant lipid metabolism, which is defined by increased absorption and abundance of lipids along with a general dependence on fatty acids (FAs). Upregulated lipogenesis involves the synthesis of triacylglycerols (TAGs) and FAs [21]. There also seems to be a deregulation of mitochondrial oxidation, elongation, and desaturation. The evidence of a "lipogenic phenotype" linked to the development and progression of CRC is also supported by the upregulation of lipid subclasses such as ceramides (CERs), sterol esters (SEs), phosphatidylglycerols (PGs), phosphatidylcholines (PC), phosphatidylethanolamines (PEs), and phosphatidic acids (PAs) [42]. Furthermore, according to Liu et al. [26], FAs and ethanolamine plasmalogens may have been the first CRC diagnostic biomarkers in plasma. Moreover, TAGs have been discovered to be the primary lipid markers that are disrupted by the advancement of CRC [24].

Over the past ten years, MALDI-IMS investigations have been the primary source of evidence about alterations in the lipid composition of tumors connected to malignancy [2]. Specifically, the composition and distribution of hundreds of ion signals within cells and tissues may be uncovered by using MALDI-MS and MALDI-IMS. In fact, a number of studies [29] have employed similar techniques to characterize the lipid profile in a range of cancer forms, including CRC. Shimma et al. [47] discovered region-specific lipid profiles in relation to CRC-derived liver metastases, while Thomas et al. [53] discovered that a panel of lipid-based indicators was both up- and down-regulated in these types of metastases. Thus far, no particular lipid signatures in PM samples have been identified using MALDI-MS and MALDI-IMS-based techniques.

Here, we attempted to concentrate on three key objectives: (1) Determine whether PDO from PM patients share lipidomic features using MALDI-MS; (2) Determine whether PDO features are present in cancerous and absent in healthy PM tissues using MALDI-MS and MALDI-IMS analysis; and (3) Investigate possible correlations between PDO features and PM histological features when using MALDI-IMS analysis.

## Materials and methods

### Chemicals and materials

All solvents (ULC-grade) were purchased from Merck (Italy) unless stated otherwise. 9-Aminoacridine (9AA) was purchased from Sigma-Aldrich (Italy). ITO glass slides were obtained from Bruker Daltonics GmbH (Bremen, Germany). Chloroform and ethanol (HPLC grade) were purchased from Sigma-Aldrich (Italy). Methanol (LC–MS grade) was from Fisher Scientific (Italy).

### Human tissues and PM-derived PDOs

Peritoneal tissues were collected from patients with PM who underwent surgical resection at the Peritoneal Malignancies Unit of Fondazione IRCCS Istituto Nazionale dei Tumori di Milano. The study was approved by the Institutional Review Board (134/13; I249/19) and was conducted in accordance with the Declaration of Helsinki, 2009. Written informed consents were acquired. The six patients (PT21_14, PT19_01, PT19_03, PT21_11, PT-R1T, and PT19_06) whose tumor samples were acquired and subjected to MALDI-IMS analysis have their clinical features listed in Supplementary Table 1. By using MALDI-IMS, it was feasible to analyze normal peritoneum tissue for patients PT21_14 and PT21_11. Six additional normal peritoneum samples from a different group of PM patients (six patients) were subjected to MALDI-MS analysis. Every normal tissue was removed over ten centimeters away from the metastases. One part of the metastatic tissue (1 cm in diameter) was placed in ice-cold phosphate-buffered saline (PBS; ThermoFisher Scientific) containing gentamicin (50 ng/ml, ThermoFisher Scientific) and amphotericin B (50 ng/ml, ThermoFisher Scientific) for the generation of PDOs, while a second specimen was frozen in liquid nitrogen for molecular and histopathological analyses. PDOs were established as described in Varinelli et al*.* [59]. PDOs were successfully established from metastatic lesions of patients PT19_03, PT19_06 and PT21_11. PDO cultures used for the MALDI-MS study was seeded on 24-well plates and cultured to produce roughly 250 PDOs per well. After that, PDOs were mechanically removed from the Matrigel by adding a few µl of Cell Recovery Solution (Corning) to the cell culture plate. PDOs from six wells (about 500,000 cells) were combined into a single sample for lipid extraction after being twice rinsed in ice-cold PBS.

### Lipid extraction from PDOs

Lipids from PDO cultures or PM-derived tissues were extracted using Bligh and Dyer’s method [4] as described in Ghislanzoni et al. [11]. 300 μL of chloroform/methanol (2:1, v/v) was added to the cellular pellet. The suspension was then mixed for 15 min at 1400 rpm and sonicated for 4 min. Deionized water (100 μL) was added to separate the organic phase from the aqueous phase, and the suspension was mixed for 1 min before centrifuging at 3000 g for 5 min at 20 °C. The organic phase (lower layer) containing lipids was collected in a clean tube and dried in a SpeedVac centrifuge. Lipids were then resuspended in an appropriate volume of 2-propanol/ACN (60/40, v/v) and sonicated. Next four replicates of 1 μL of the mixture were layered on the indium tin oxide coated slides (ITO slide, Bruker Daltonics), dried in a desiccator for approximately 60 min, and then stored at -80 °C until use.

### Tissue samples processing

Sample preparation for MALDI analysis was performed starting from the patient's frozen biopsy stored at -80 °C. Frozen PM-derived samples were cut into 10 µm-thick slices using a cryostat (Leica Instruments GmbH). For IMS analysis, frozen tissue sections were mounted on ITO slide (Bruker Daltonics), dried in a desiccator for approximately 60 min, and then stored at -80 °C until use. At the same time, other sections were cut and collected into vials for tissue homogenization and lipid extraction using the Bligh and Dyer method [4]. Lipids were resuspended in an appropriate volume of 2-propanol/ACN (60/40, v/v) and sonicated. One μL of lipid suspension was deposited on ITO glass slides, performing replicates.

### MS analyses

For every MALDI analysis, the 9-AA matrix was solubilized in 2-propanol/ACN (60/40, v/v) to a final concentration of 10 mg/mL and applied onto the ITO glass slides using a TM sprayer (HTX Technologies, Chapel Hill, NC). For its application, the nozzle temperature was set to 80 °C, the pressure to 10 psi, and the flow rate of the LC pump to 0.12 mL/min. After matrix application, samples were left in the desiccator until spectra were acquired. MS analysis was performed using an UltrafleXtreme MALDI MS instrument equipped with a 1 kHz SmartBeam II Nd:YAG/355 nm as an ionization source (Bruker Daltonics). Spectra were obtained in reflector negative mode within the m/z range of 600 to 1.600. An internal calibration was performed by setting the peak of the deprotonated PA (18:0–18:1) at m/z 701.50, ST (d18:1-C16:0) at m/z 778.52 and PI (18:1–20:4) at m/z 885.56. Automatic acquisition was based on pixel-by-pixel mode, guided by FlexControl 3.4 and FlexImaging 4.1 software. Single mass spectra were acquired by applying 200 laser shots in continuous raster mode over a distance of 20 µm and scanning areas of ~ 4 mm^2^. Data were subsequently analyzed in SCiLS lab version 2024 (Bruker Daltonics) by “processing methods” for peak picking, baseline subtraction, and smoothing operations. Centroid algorithm was used for peak picking. All spectra were normalized to the total ion count (TIC).

For IMS on tissue sections, 200 laser shots were summed to generate a representative spectrum for each pixel, with the digitizer sampling rate at 1.25 GS/s. The spatial resolution for tissue section imaging was set in the range of 50 to 70 μm. Software from Bruker Daltonics (FlexControl, FlexImaging, FlexAnalysis, and SCiLS) was used for spectra acquisition and analysis. Data analysis was done by “processing methods” for peak picking, baseline subtraction, and smoothing operations. The centroid algorithm was used for peak picking. Following MALDI-IMS, tissue sections were washed with methanol for 30 s to remove the 9-AA matrix and then stained with hematoxylin and eosin (H&E). The H&E-stained slides were then acquired at high resolution (40X) using the Aperio Scanscope Slide Scanner, imported into QuPath software, and co-registered with the acquired MALDI-IMS data using SCiLS Lab Software.

The MALDI-LIFT™ (MS/MS) fragmentation technique was employed to produce ions related to headgroup and fatty acyl chain loss, enabling the determination of the lipid class, as outlined in a previous study by Suckau et al. [50]. A preliminary determination of lipids was made by searching the LIPID MAPS database (https://www.lipidmaps.org) with a tolerance of ± 0.2 Da. Both the homogenate samples and tissue slides were subjected to direct application of the LIFT™ method. In order to account for the fairly limited resolving power of around 5000 at 885.56 (with a mass accuracy of < 0.2 Da), tentative assignments were made only if the proposed lipid species were previously described.

### Statistical analyses

SCiLS software was used for statistical analysis of MS and IMS data. Simultaneous preprocessing of all data sets was performed to ensure better comparability between the sample sets. Imported data were pre-processed by convolution baseline removal (width: 20) and TIC normalization. The spatial bisecting K-means algorithm (correlation distance) was used to calculate the spatial dependency between locations and to determining the similarity of mass spectra at each location. To define common molecular features among the sample sets, different unsupervised multivariate classification methods for mass spectra were applied: principal component analysis (PCA) was performed to determine the largest difference between PM and control samples as previously described [13]. Additionally, probabilistic latent semantic analysis (pLSA) was applied to determine differences between the histological features and their corresponding characteristic mass spectra. Receiver operating characteristic analysis (ROC) was used to assess the quality of all values within specific regions of interest (ROI) to discriminate between intra-tumor areas. For this method, the number of spectra in the ROIs of both groups was approximately the same.

## Results

### MALDI-MS analysis of lipids derived from PDOs

We analyzed the MALDI-MS lipid profiles of PDOs from the metastatic tissues of three PM patients, identified as PT19_03, PT19_06, and PT21_11. The mass spectra were acquired in negative mode. Lipids were categorized as: 1) glycerophospholipids (GPLs), including phosphatidylcholines (PMs), phosphatidylethanolamines (PEs), phosphatidylserines (PSs), phosphatidic acids (PAs), phosphatidylinositols (PIs), phosphatidylglycerols (PGs), and cardiolipins (CLs) [31]; 2) sphingolipids and glycosphingolipids (GSLs) [41], and 3) glycosylphosphatidylinositols (GPIs) as the glycan-phosphatidylinositol anchor precursors (GPI-AP) [33].

**Fig. 1:**
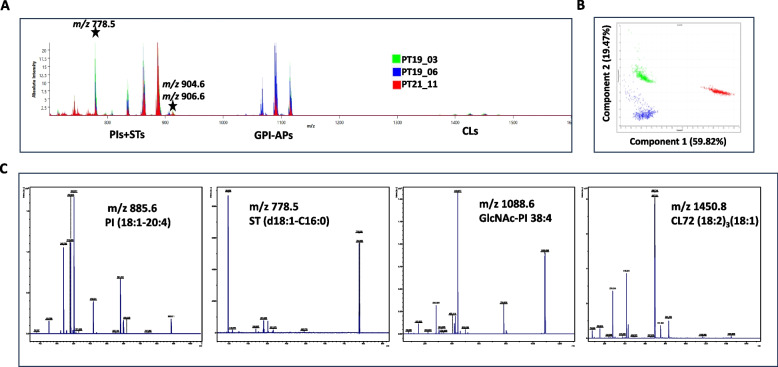
Negative ion mode MALDI-MS analysis of the lipid extracts of three PDO cell lines derived from PT19_03, PT19_06 and PT21_11 patients. **A** Lipid profiles acquired in negative ion mode by automatic acquisition. Lipid species can be grouped in two main ranges: the *m/z* range 700–900, where GPL and GPS species are present, and the m/z range 1100–1500, where minor peaks compatible with GSLs and CLs species are visible. **B** Two-dimensional principal component analysis (2D PCA) scores plot of the individual PDO cumulated spectra (525, 483 and 462, respectively) using 131 aligned peaks. Two principal components (PC) explained 79.28% of the variance of the data. In (**C**), we show the fragmentation pattern of ions with *m/z* 885.6, 778.5, 1088.5, and 1450 obtained by MS/MS analysis. *m/z* 885.6 corresponding to PI 38:4 was fragmented into the characteristic product ions with *m/z* 419.2, 283.1, and 240.8; *m/z* 778.5 was fragmented into ions with *m/z* 96.9 (hydrogen sulfate ion), *m/z* 258.7 (dehydrogenated galactose-sulfate) and *m/z* 240.7 (dehydrated galactose-sulfate), ions known to be representative signals of the ST head group and corresponding to ST d18:1-C16:0. The *m/z* 1088.6 ion corresponding to the GlcNAc-PI 38:4 species [15] was fragmented into product ions with *m/z* 443.9 (GlcNAc-myo-inositol-1,2-cyclic phosphate) and its dehydration product at *m/z* 425.7. These two ions are characteristic of the negative ion GlcNAc-PI spectra. Peaks at m/z 78.7, 152.7, and 282.9 are (phosphate), (glycerol-cyclic phosphate), and (stearate), respectively. *m/z* 1450.8 was fragmented into product ions with *m/z* 831.7, 751.7, 695.6, 697.6, 415.2, 417.5, 281, and 279 typical fragments of CL72:7, with the most intense peaks corresponding to linoleate (*m/z* 279), monoacylglycerol phosphatidate (*m/z* 415.2), and diacylglycerol phosphatidate (*m/z* 695.6)

PDO profiles revealed many similarities (Fig. 1A). Nonetheless, a distinct grouping of the three PDOs was seen in the scatter plot produced by the PCA-based unsupervised data processing of the 131 aligned ion peaks (Fig. 1B). A clear separation between the PDOs from PT21-11 patient and the other two PDO cell lines from PT19_03 and PT19_06 patients was observed along the first component.

The peaks at *m/z* 807.6, 833.6, 835.6, 861.6, 863.6, and 885.6 were attributable to PI species 32:1 (16:0–16:0), 34:2 (16:1–18:1), 34:1 (16:0–18:1), 36:2 (18:1–18:1), 36:1 (18:0–18:1), and 38:4 (18:0–20:4), respectively (Fig. 1 and Suppl. File 1). The 861.5 PI species that contain two monounsaturated FAs (MUFAs) unexpectedly prevailing over the 885.6 PI species containing the polyunsaturated FAs (PUFA), C20:4 (AA) in all PDO samples. The peaks corresponding to the main GSL sulfates (STs) (peaks at *m/z* 778.6, 904.6, and 906.6) (Fig. 1A) were abundantly present in all PDO cell lines. Clusters of CLs and GPI-APs at different intensities were represented in all three PDO lines.

ST species varied in terms of sphingoid base and FA composition. The species we detected were mainly galactosyl STs (SM4s, [17]), rich in d18 sphingoid base and C16:0, C24:0, and C24:0-OH FA chains (Fig. 1A, C and Suppl. File 1).

Among GPI-APs, the most intense peak was at *m/z* 1088.6, which is normally the predominant species containing C20:4 and the stearic acid with a saturated C18 chain (Fig. 1A, C and Suppl. File 1).

Peaks that corresponded to PIs, STs, and GPI-APs dominated the spectra from the three PDO lines. Each of these lipids plays a crucial role in determining the membrane's structural microdomains and how it behaves. Higher PI (18:0–18:1) levels in PIs may help PDO cells since they may make them more resilient to metabolic stress [55].

### Tissue classification based on lipid profiles

**Fig. 2:**
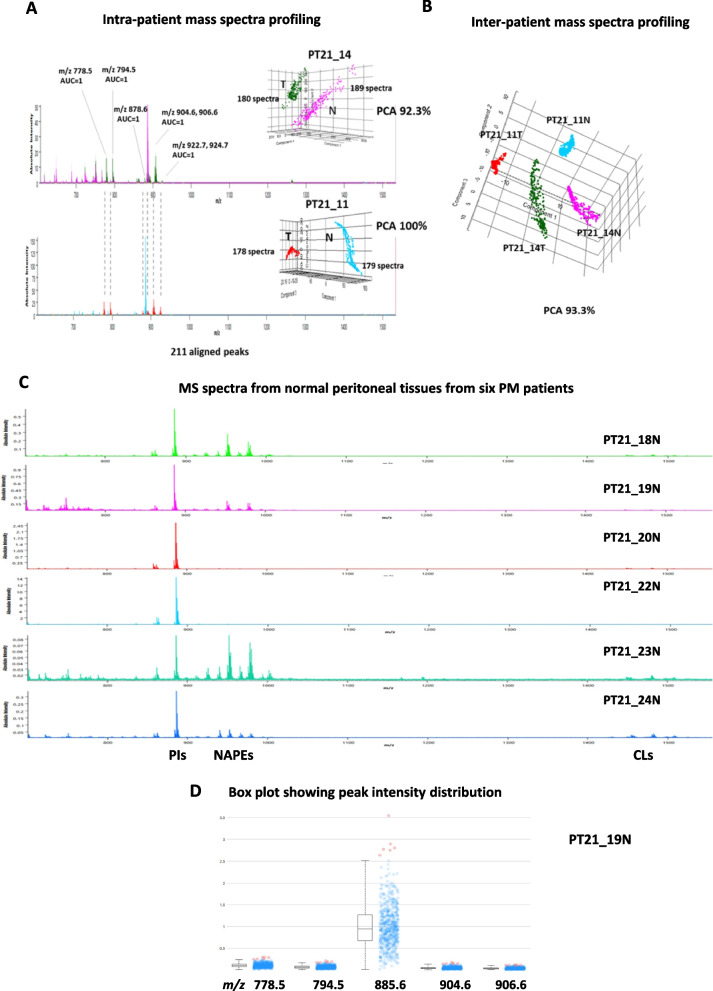
MALDI-MS analysis of PM *vs* matched healthy peritoneum from PT21_11 and PT21_14 patients. **A** Intra-patient mass spectra peaks of PM lesion *vs* healthy peritoneum with the AUC values of the most discriminative peaks. **B** 3-dimensional plot of PCA scores of inter-patient mass spectra peaks originating from healthy peritoneum and tumor tissue. A percentage explaining the variance between samples is shown. **C** MS spectra from normal peritoneal tissues from six PM patients. **D** The peak intensities distributions in solubilized PT21_19N tissue are depicted through box plots that match *m/z* values of certain STs (778.5, 794.5, 904.6, 906.6) and PI 38:4 (885.6)

We investigated the potential for identifying PDO lipid characteristics in the tumor cell regions of clinical PM samples, especially those related to ST species. We first looked at the lipid profiles of homogenates made from PM samples (PT21_11T and PT21_14T) from two patients, as well as the lipid profiles of nearby healthy peritoneal tissues (PT21_11N and PT21_14N). Figure 2 displays the averaged MALDI mass spectra of PM samples along with the matched healthy peritoneum. It was feasible to identify with precision the profiles of PM and healthy peritoneum in both patients after applying unsupervised PCA to the spectra data (Fig. 2A). This enabled us to assess if the analysis was sensitive enough to identify tumor-containing tissues.

In terms of ST species, the AUC value supported the hypothesis that STs are characteristics of tumor cells in PDOs and in PM tissues. In fact, ST-related peaks: at *m/z* 778.5 (d18:1-C16:0), 794.5 (d18:1-C16:0-OH), 878.6 (d18:1-C22:0-OH), 904.6 (d18:1-C24:1-OH), 906.6 (d18:1-C24:0-OH), and 922.7–924.7 (t18:0-C24:0, t18:0-C24:1)) could distinguish between tumor and non-tumor tissues (Fig. 2A). Additionally, the complete separation of the PCA-based grouping demonstrated the uniqueness of the peritoneum and PM lesions (Fig. 2B).

In order to investigate the existence or lack of sulfatide species in normal peritoneal tissues further, we used MALDI-MS to analyze normal peritoneum tissue from six different PM patients. Normal peritoneum tissue from a new series of PM patients, collected distal to metastatic lesions, was solubilized and subjected to MALDI-MS analysis (Fig. 2C and D) in an effort to explore the presence of STs. It is evident from a comparison of the six profiles that there are numerous commonalities among the lipid profiles of (Figs. 2C), notwithstanding their imperfect overlap. The primary peaks in the mass spectra cells can be seen in the major GPLs range. Different PI species are given to the signals at m/z 835.6, 863.6, and 885.5. Additionally, peaks potentially corresponding to N-acylphosphatidylethanolamine (NAPE) species and minor peaks corresponding to CLs species were found in the lipid profiles. The boxplots of the PI 38:4 and STs peak intensities in the peritoneal tissue of the PT21_19 patient is shown in Fig. 2D, and they may be used as a representative example for the other peritoneal tissues under consideration. We can infer from these results—which are still based on a small sample size—that ST species is either absent or weakly detectable in the normal peritoneal tissues of PM patients.

### Spatial segmentation of lipid species in PM lesions

**Fig. 3:**
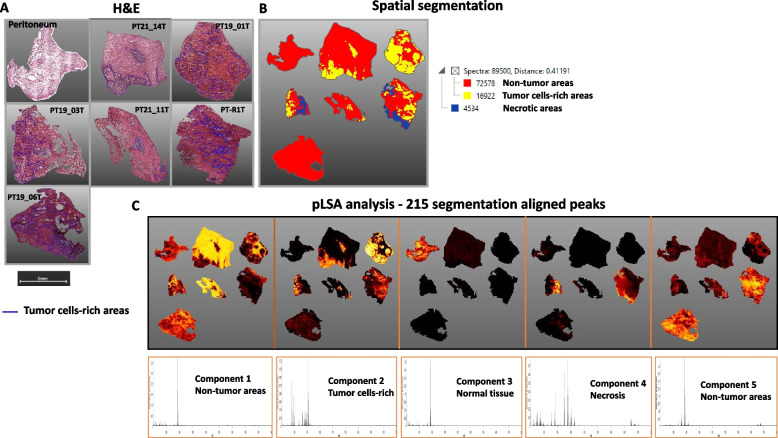
MALDI-IMS of tissue sections. **A** Sections from a normal peritoneum and PM lesions from six patients, stained with H&E for sections overlapping with the MALDI-IMS image. Blu lines surround tumor cells-rich areas (annotated by the reference pathologists). **B** Segmentation map analysis of MALDI-IMS spectral data. Assigned areas per spectral similarity are indicated with color generated by bisecting k-means clustering based on 215-aligned peaks and dendrogram with the respective correlation distances (numbers) representing non tumor cells-rich areas (in red), tumor cells-rich areas (in yellow) and necrotic areas (in blue). **C** Unsupervised multivariate analysis and correlation with sections’ regions. The unsupervised multivariate analysis was performed on the single pixels using pLSA based on 215-aligned peaks with deterministic initialization algorithm; week denoising and TIC normalization were performed

Six PM lesions, derived from six different PM patients (PT21_14T, PT19_01T, PT19_03T, PT21_11T, PT_R1T, PT19_06T), and one healthy peritoneum section from patient PT21_14 (PT21_14N), were subjected to a histology-directed MALDI-IMS analysis to determine whether PDOs lipid features could distinguish tumor cell components from stromal components and healthy peritoneum. For each tissue, we show the hematoxylin–eosin (H&E) staining after the removal of the 9AA matrix (Fig. 3A) that highlighted the complex and heterogeneous morphologic structure of PM lesions and the lipid-based segmentation map, obtained via bisecting k-means clustering by SCiLS Lab software (Fig. 3B), that efficiently captured heterogeneity in a spatially resolved manner.

It clearly produced the distinction between tumor cell–rich areas (yellow) and the adjacent stromal areas (red) and necrotic areas (blue). PT21_14N, the healthy peritoneum, clustered with stromal regions (red). This suggests that while areas of tumor tissues rich in stroma or poor in tumor cells resemble normal normal peritoneum tissue, areas of tumor tissues rich in tumor cells show similar histo-molecular spectrum properties.

Software from SCiLS Lab was used to analyze the spectra in order to understand the changes in lipid profiles. The spectra were then separated using pLSA, which associates MS data with specific areas and makes lipid fingerprint deconvolution easier. We considered 215 peaks that were present in all samples. A total of five components turned out to be the best number for explaining sample variance. Figure 3C shows the distribution of pLSA scores for healthy peritoneum and PT-tumor samples and Fig. 3D-related spectra components. The segmentation outcomes are cross-corroborated by these results (Fig. 3A and B). Components 1 and 3 define peritoneal tissue; component 1 is distributed in 4 of the 6 PM lesions and reflects a stroma type where PIs, especially PI 38:4, and gangliosides (GGs) are predominant, while component 3 depicts the lipid-specificity of the single healthy tissue.

Component 2 characterizes regions of the tissue with a high concentration of tumor cells and a high intensity of ST ion species. The ST-related peaks, which were hallmarks of pLSA component 2, were exclusively seen in tumor tissue areas. They had *m/z* values of 778.5 (d18:1-C16:0), 794.5 (d18:1–16:0-OH), 878.6 (d18:1-C22:0-OH), 904.6 (d18:C24:1-OH), 906.7 (d18:1-C24:0-OH), and 922.6–924.6 (t18:0-C24:0-OH, t18:0-C24:1-OH).

**Fig. 4:**
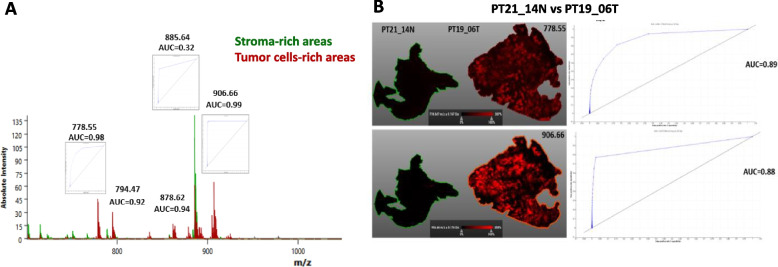
Univariate analysis of MALDI-IMS data determining single lipids is the most discriminative between stroma-rich and tumor cells-rich areas. **A** ROC analysis (AUC values) testing the power of ST-related peaks to distinguish between normal peritoneum tissue or regions rich in stroma and areas rich in tumor cells. Note it is shown a portion of the spectrum containing 215-aligned peaks that was used for automatic segmentation showed in Fig. 3. **B** STs discriminate between PT19_06 (PM sample) and normal peritoneum. PT19_06 shows discriminant STs (d18:1-C16:0 and d18:1-C24:0-OH) ion intensity when compared to normal peritoneum

Based on the AUC value, discrimination ability was calculated. An optimal discriminating capacity (AUC > 0.9) of ST species, which are defined by *m/z* 778.5, 794.5, 878.6, and 906.7, was found by ROC curve analysis on data subsets that include both tumor and stroma areas that are specified by a segmentation map (Fig. 4A). Using the identical pLSA, patient PT19_06 did not have this outcome. Nevertheless, weak ion intensity for *m/z* 778.5 and 906.7 was seen in a two-way comparison pLSA experiment comparing PT21_14 (healthy peritoneum) (Fig. 4B).

**Fig. 5:**
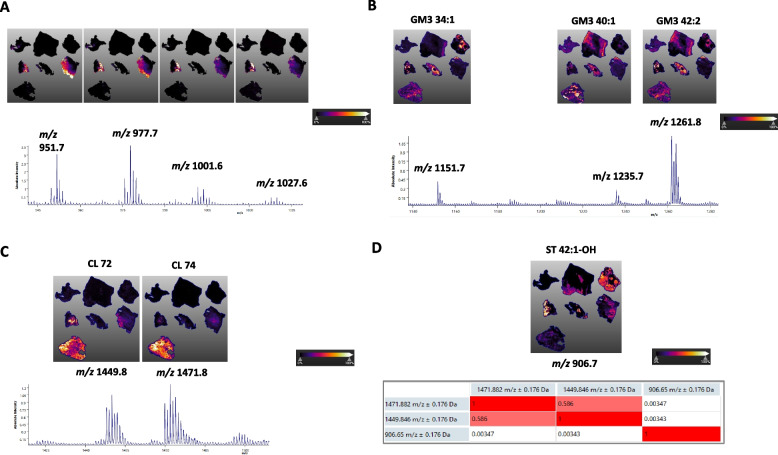
Spatial localization of selected lipid species. **A** NAPEs distributions in necrotic areas (N); **B** GGs-distributions in stroma areas; and CL72 and CL74 distributions in **C** together with their inverse correlation with ST 42:1-OH (at 906.7, ST(d18:1-C24:0-OH)) in **D**

pLSA 3 represents lipids that are significantly upregulated in the healthy peritoneum (Fig. 3). The pLSA component 4 matched the necrotic regions in two patients and also contained interesting species at *m/z* 951.7, 977.7, 1001.6, and 1027.6, potentially corresponding to NAPE species (Fig. 5A, Suppl. File 2). pLSA component 5 included PI, GG, and CL species, mainly localized in the stromal areas (Fig. 5B).

Four major CL clusters were observed in components 4 and 5, most of which had multiple individual CL species at several isobaric masses (Fig. 5C). An interesting inverse correlation for spatial distribution was noted for CL and ST species (Fig. 5D). GPI-APs that were particularly represented in PDOs (see Fig. 1) were not confirmed as biomarkers in the PM lesions.

### ST localization was limited to tumor cells-rich areas

**Fig. 6:**
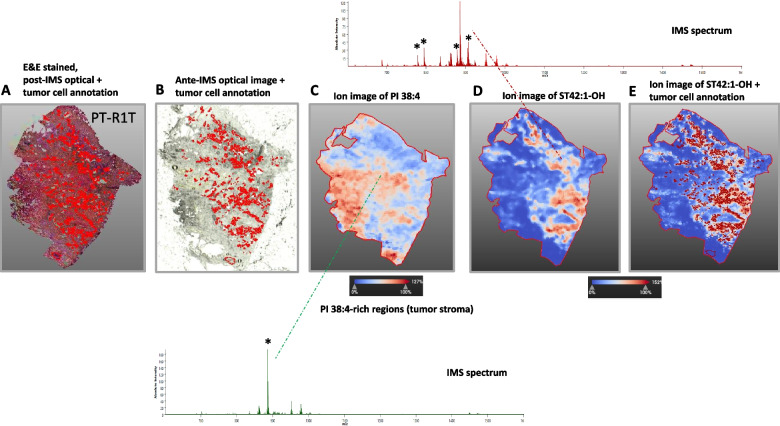
PT-R1T tissue section images showing the QuPath markup image. **A** Images correspond to H&E-stained section; **B**-**E** optical and ion images showing the spatial distribution of PI 38:4 and ST 42:1-OH. Red mask indicates tumor cells-rich areas annotated by the pathologist using QuPath. Representative spectra for PI 38:4-rich areas and ST 42:1-OH-rich areas are shown. Ions corresponding to different STs and PI38:4 are indicated by asterisks

Figure 6 shows H&E-stained microscopic images of the PT_R1T sample that can help in visualizing the morphology of the tissue and therefore can help in the identification and differentiation of tumor areas (marked in red) in the tissue. The same areas were overlaid with an optical image. The expression pattern of two differentially expressed ions at *m/z* 885.6 (PI 38:4) and 906.7 (ST 42:1-OH) is shown. The visualization allows a straightforward correlation of the expression pattern with the tissue morphology of PT_R1T slide (Fig. 6). While the ion at *m/z* 885.6 (PI 38:4) is most intensely distributed in the stroma, *m/z* 906.7 (ST 42:1-OH) is more intense in tumor cells’ areas. Asterisks indicate ions corresponding to other STs colocalized with ST 42:1-OH species.

The PT-R1T sample's H&E-stained microscopic pictures, shown in Fig. 6, can aid in the identification and distinction of tumor regions (highlighted in red) in the tissue by allowing one to see the tissue's morphology. The optical picture was superimposed over the same regions. Two differently expressed ions at *m/z* 885.6 (PI 38:4) and 906.7 (ST 42:1-OH) are displayed along with their expression patterns. A simple association between the expression pattern and the tissue shape of the PT-R1T slide can be made thanks to the visualization (Fig. 6). The stroma has the most intensive distribution of the ion at 885.6 (PI 38:4), but the regions around tumor cells have a higher intensity of *m/z* 906.7 (ST 42:1-OH). Ions corresponding to different STs are indicated.

## Discussion

Precision medicine is applied in oncology to better identify which patients will benefit from a specific treatment in order to improve their clinical outcome [45]. Genomic platforms are the preferred strategy for identifying genetic mutations that may be suitable for predictive therapy. Although, this approach results in increased response rates [8, 40, 61], it is not yet fully satisfactory. For this reason, it is of great importance to combine a drug screening on patient-derived tumor cells. PDOs are widely used to examine the effects of thousands of drugs and select the most suitable ones [20, 60] as they have been shown to better recapitulate key aspects of tissue composition, including tumor architecture, heterogeneity, function, remaining genetically stable in culture [16].

PM is a very low-targetable tumor disease and there is an urgent need to develop more effective therapies. Several groups, including ours, are exploring the possibility of using PDOs for drug discovery [3, 10, 27, 56, 59].

Our methodology was based on the already proven concept that PM undergo profound alterations in lipid homeostasis due to reprogramming of metabolism, which in turn affects signaling pathways [23, 36]. We developed screening modalities to profile lipids in PDOs and naïve biopsies using MALDI-MS and MALDI-IMS [49]. Consistent with our methodology, we observed lipid species including PAs, PIs, GPI-APs, STs, GGs and CLs and identified lipid signatures peculiar to PDOs and PM biopsies.

The key lipid classes identified emerged in all three PDOs, despite the different mutational status and growth conditions. Specifically, the spectra of the three PDO lines showed commonalities in the signatures of PI, ST and GPI-AP precursor species, but showed remarkable differences in the CL region.

While GPI-APs accumulation was not observed in the clinical samples, the expression of specific STs was evident in both PDOs and PM biopsies with a similar pattern. It should be considered that the culture method of PDOs may influence chemical and mechanical properties by producing effects on PDO growth, development, or morphology [39].

In our MALDI-IMS experiments on PM and normal peritoneum, lipid patterns are clearly identifiable. The pLSA component 1 is primarily detecting variations within the stroma areas of tumor tissues from various patients. It is distinguished by a high intensity of PI that contains AA, specifically PI (18:0–20:4). According to Kawashima et al. [18], PI (18:0–20:4) was mostly found in stromal areas surrounding the cancer cells and had strong concentrations that varied across individuals. According to Hardwick et al. [14], AA (as a precursor of eicosanoids) and the PI cascade are related to inflammation and insulin resistance.

For every tumor specimen, the pLSA component 2 correctly identified the ST-positive regions. Tumor cells contained ST species, not the healthy peritoneum or the stroma surrounding the tumor cells.

In the healthy peritoneum, pLSA component 3 was found to include high levels of PI (18:0–20:4) and PA species, which are known to be powerful cell signaling molecules [7] and [25]. Furthermore, PA species are the main precursors of all GPIs, including PM, PE, and PS species [48]. The necrotic regions' diversity was recorded by the pLSA component 4, which was mostly represented by lipids that could have been NAPE species are synthesized by acylation of the free amine group of ethanolamine-containing glycerophospholipids can be defined as signaling lipids [30, 34, 44, 62].

The variation related to the GG-enriched regions of the stroma, such as monosialoganglioside GM3 (d18:1–24:1) and GM3 (d18:1–16:0), was detected by pLSA component 5. It is noteworthy that cancer cells can release GGs into the tumor microenvironment, where they may stimulate the growth of tumors and function as an anti-tumor immunity [57]. Given that the majority of GG species exhibit strong immunosuppressive properties, it is possible to imagine them as checkpoint molecules that are released to disrupt continuous immunosurveillance. This is interesting because stromal areas are where we found these lipid species.

ST species specifically identified tumor cells in both PDOs and clinical samples. Galactose from UDP-galactose is added to ceramides with a final 3-O-sulfation alteration to create STs, which are sulfated GSLs. STs are multifunctional chemicals that have several roles in the neurological system, immunological system, bacterial and viral infections, hemostasis and thrombosis, insulin production, and hemostasis. Accordingly, illnesses arise because of aberrant metabolism or changed ST expression [52]. In several human malignancies, such as renal cell carcinoma, colon and lung adenocarcinomas, and ovarian cancer, where they have been demonstrated to facilitate metastasis, STs may accumulate up as a result of increased galactosylceramide sulfotransferase (Gal3ST1) increased activity [38]. Notably, ST expression and concentration are raised in renal cell carcinoma, and this has been mechanistically connected to ST upregulation by HIF1α [52].

It is not clear what regulates the metabolism levels of sulfatides inside cells. Research shows that peroxisome proliferator-activated receptor α (PPARα) activation increases the synthesis of sulfatides in the mouse liver by enhancing the activity of cerebroside sulfotransferase (CST), a key enzyme in sulfatide production [32]. Another research study indicates that PPARα controls both the beginning and end stages of the sulfatide production process [63]. Therefore, it has been suggested that activating PPARα can help cells safeguard against and reduce the negative impacts of both fatty acids and sulfuric acid by increasing sulfatide production. In the same vein, clofibrate and fenofibrate are classic PPARα agonists which can increase sulfatides levels [19]. MK886, an inhibitor of PPARα, induced a decrease in serum sulfatides levels in skin cells from patients with very long-chain acyl-CoA dehydrogenase deficiency and excessive fatty acid accumulation activated PPARα and increased sulfatides levels [63]. Thus, several lines of evidence suggest that PPARα activation is a crucial regulator of sulfatides levels.

Several highly expressed ST species, including hydroxylated species that co-localized with ST enrichment zones, were found in PM by our research. Like other GSLs, STs can be hydroxylated and have a varied structure with varying acyl chain and ceramide moiety lengths. Although the precise function of fatty acid 2-hydroxylation is unknown, it seems to have an impact on membrane fluidity [12]. Furthermore, STs containing the long- and short-chain isoforms (C16:0 and C24:0, respectively) are typically detected, the former is associated with the maturation of insulin [5], while the latter is connected to the inhibition of the immune system [6]. Despite these initial observations, future studies are necessary to further explore ST’s pathological role in the PM.

## Conclusion

Numerous recent studies and clinical procedures have shown that patients with intraperitoneal metastases can benefit from local surgery, such as HIPEC, which can reduce symptoms and improve quality of life, despite the widespread belief that metastatic tumors cannot be surgically treated. The results of recent investigations have shown that abnormal lipid metabolism is one of the key mechanisms underlying tumor dissemination. Therefore, treating abnormal lipid metabolism may lower the incidence of tumor metastasis. It is assumed that metastases may not form at all or may occur less frequently if one or more metabolic reprogramming processes are blocked during the tumor metastasis process. Hence, developing medications that focus on these specific targets could benefit individuals with advanced forms of cancer.

PDO lipid components are identified in PM patients' metastatic lesions by using MALDI-MS and MALDI-IMS. The PDO lipids found in cancerous metastatic lesions are common to peritoneal tumors and/or are barely detectable in normal tissues. These tumor specific lipid features are very useful in determining the spatial organization and phenotypes of tumor cells in tissues, whereas traditional techniques such as histological and immunofluorescence imaging are restricted to a few molecular markers.

ST buildup in individual PM lesions is described by our proof-of-principle study that makes use of the special application of MALDI-MS and MALDI-IMS. If ST accumulation will be confirmed in a larger cohort of patients, a novel and attractive pathway for the metabolic targeting of PM metastatic growth will open up. Therefore, this feature of tumor cells may facilitate the development of novel drugs that specifically target cancer cells.

ST accumulation is common to both metachromatic leukodystrophy and multiple sulfatase deficiency. Numerous approaches are being tested in clinical trials to decrease ST accumulation, which is predominantly regulated by two enzymes: ceramide galactosyltransferase (CGT or UGT8) and galactose-3-O-sulfotransferase (CST or Gal3St1). Currently, trials are underway to evaluate initial compounds targeting enzymes that could also have potential in cancer treatment. Additionally, further investigation is needed in clinical trials regarding the signaling pathway regulated by PPARα.

### Supplementary Information

Supplementary Material 1.

Supplementary Material 2.

Supplementary Material 3.

## Data Availability

The data that support the findings of this study are available on request from the corresponding author.

## Abbreviations

9AA: 9-Aminoacridine
AA: Arachidonic acid
ACN: Acetonitrile
AUC: Area under the curve
CL: Cardiolipin
CRC: Colorectal cancer
CRS: Cytoreductive surgery
FA: Fatty acid
Gal3ST1: Galactosylceramide sulfotransferase
GG: Ganglioside
GPI: Glycosylphosphatidylinositol
GPI-AP: Glycan-phosphatidylinositol anchor precursors
GPL: Glycerophospholipid
GSL: Glycosphingolipid
H&E: Hematoxylin and eosin
HIF1: Hypoxia-inducible factor
HIPEC: Hyperthermic intraperitoneal chemotherapy
HPLC: High-performance liquid chromatography
MS: Mass spectrometry
IMS: Imaging mass spectrometry
ITO: Indium tin oxide
LC–MS: Liquid chromatography-mass spectrometry
LIFT: Laser ionization fragmentation technology
MALDI: Matrix-assisted laser desorption/ionization
PA: Phosphatidic acid
PBS: Phosphate-buffered saline
PM: Peritoneal metastases
PC: Phosphatidylcholine
PCA: Principal component analysis
PDO: Patient-derived organoid
PDX: Patient-derived xenograft
PE: Phosphatidylethanolamine
PG: Phosphatidylglycerol
PI: Phosphatidylinositol
pLSA: Probabilistic latent semantic analysis
PM: Peritoneal metastases
ROC: Receiver operating characteristic
ST: Sulfatide
TAG: Triacylglycerol
TIC: Total ion count
TOF: Time of flight
UDP-glucose: Uridine diphosphate glucose
