# Supplementary figures and images for: Sulfatide imaging identifies tumor cells in colorectal cancer peritoneal metastases
Source: Cancer Metab. 2024 Jun 28;12:18. doi: 10.1186/s40170-024-00345-3 (PMC11212237; doi:10.1186/s40170-024-00345-3)

## Slide 1
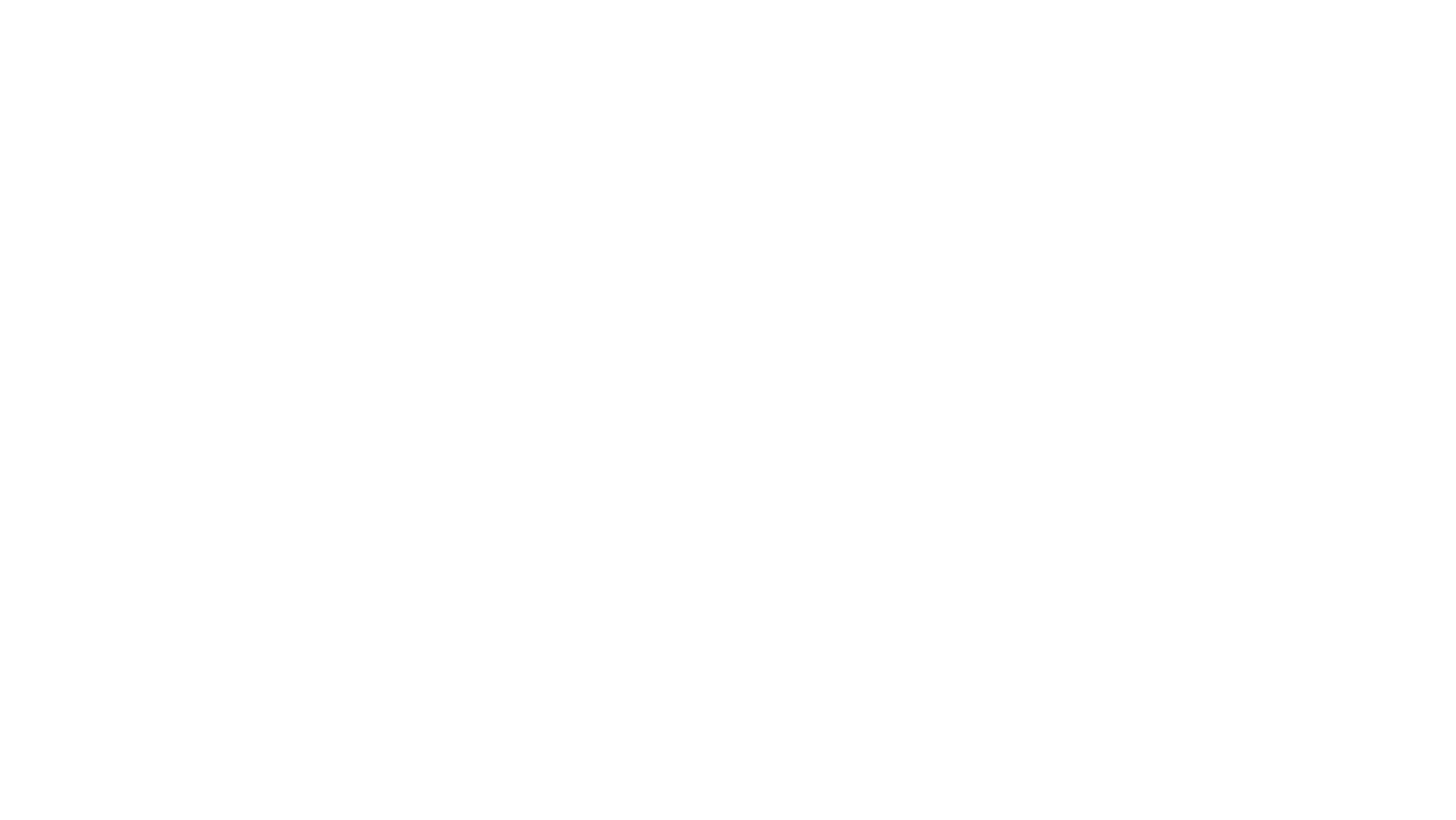

Supplement: Supplementary file 1 — Supplementary Material 1. [file 40170_2024_345_MOESM1_ESM.pptx]

## Slide 1
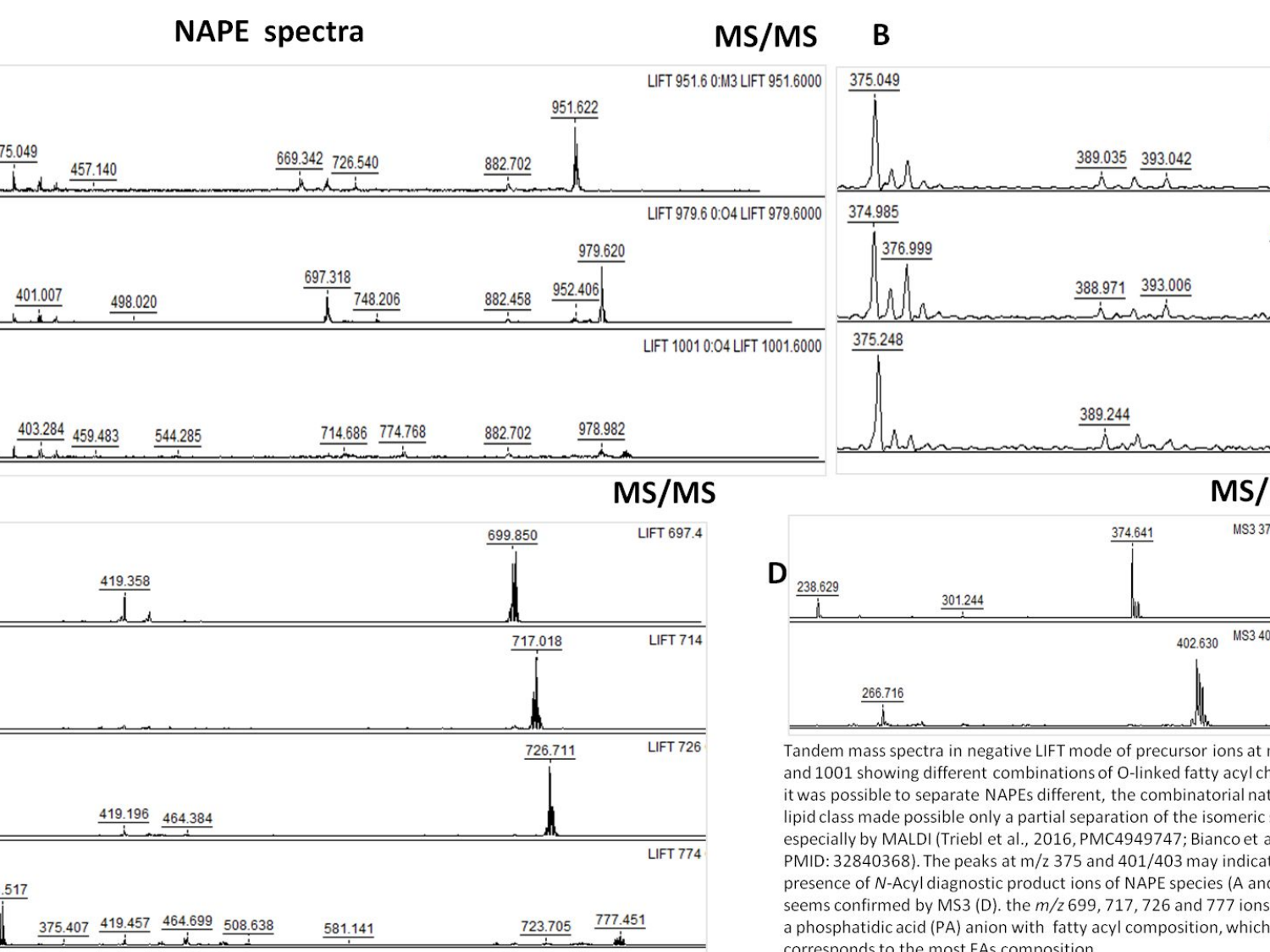

Supplement: Supplementary file 3 — Supplementary Material 3. [file 40170_2024_345_MOESM3_ESM.pptx]
