# Supplementary material for: Sulfatide imaging identifies tumor cells in colorectal cancer peritoneal metastases
Source: Cancer Metab. 2024 Jun 28;12:18. doi: 10.1186/s40170-024-00345-3 (PMC11212237; doi:10.1186/s40170-024-00345-3)
Supplement: Supplementary file 2 — Supplementary Material 2. [file 40170_2024_345_MOESM2_ESM.pptx]

## Slide 1
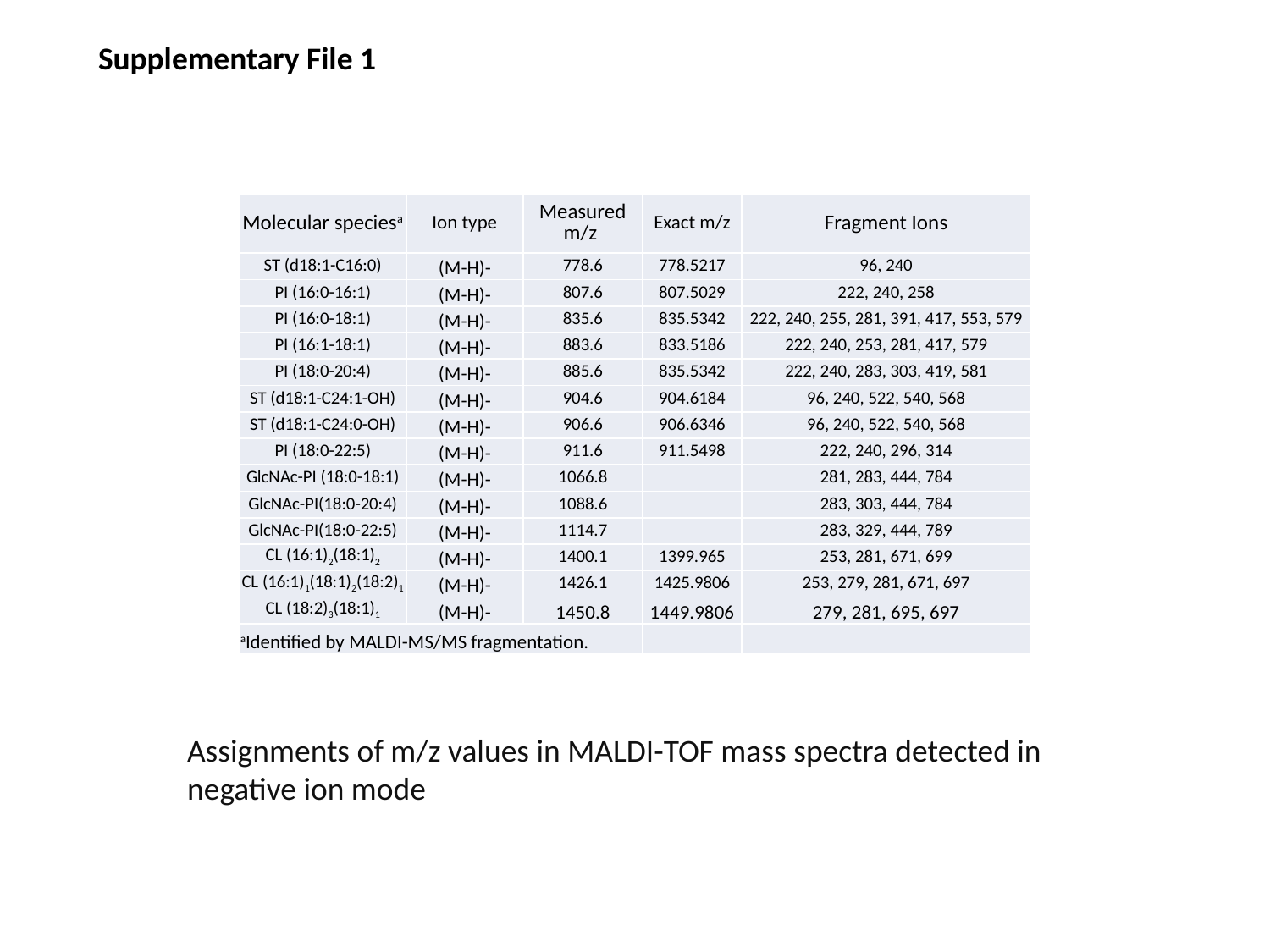

Supplementary File 1
| Molecular speciesa | Ion type | Measured m/z | Exact m/z | Fragment Ions |
| --- | --- | --- | --- | --- |
| ST (d18:1-C16:0) | (M-H)- | 778.6 | 778.5217 | 96, 240 |
| PI (16:0-16:1) | (M-H)- | 807.6 | 807.5029 | 222, 240, 258 |
| PI (16:0-18:1) | (M-H)- | 835.6 | 835.5342 | 222, 240, 255, 281, 391, 417, 553, 579 |
| PI (16:1-18:1) | (M-H)- | 883.6 | 833.5186 | 222, 240, 253, 281, 417, 579 |
| PI (18:0-20:4) | (M-H)- | 885.6 | 835.5342 | 222, 240, 283, 303, 419, 581 |
| ST (d18:1-C24:1-OH) | (M-H)- | 904.6 | 904.6184 | 96, 240, 522, 540, 568 |
| ST (d18:1-C24:0-OH) | (M-H)- | 906.6 | 906.6346 | 96, 240, 522, 540, 568 |
| PI (18:0-22:5) | (M-H)- | 911.6 | 911.5498 | 222, 240, 296, 314 |
| GlcNAc-PI (18:0-18:1) | (M-H)- | 1066.8 | | 281, 283, 444, 784 |
| GlcNAc-PI(18:0-20:4) | (M-H)- | 1088.6 | | 283, 303, 444, 784 |
| GlcNAc-PI(18:0-22:5) | (M-H)- | 1114.7 | | 283, 329, 444, 789 |
| CL (16:1)2(18:1)2 | (M-H)- | 1400.1 | 1399.965 | 253, 281, 671, 699 |
| CL (16:1)1(18:1)2(18:2)1 | (M-H)- | 1426.1 | 1425.9806 | 253, 279, 281, 671, 697 |
| CL (18:2)3(18:1)1 | (M-H)- | 1450.8 | 1449.9806 | 279, 281, 695, 697 |
| aIdentified by MALDI-MS/MS fragmentation. | | | | |
Assignments of m/z values in MALDI-TOF mass spectra detected in negative ion mode

## Slide 2
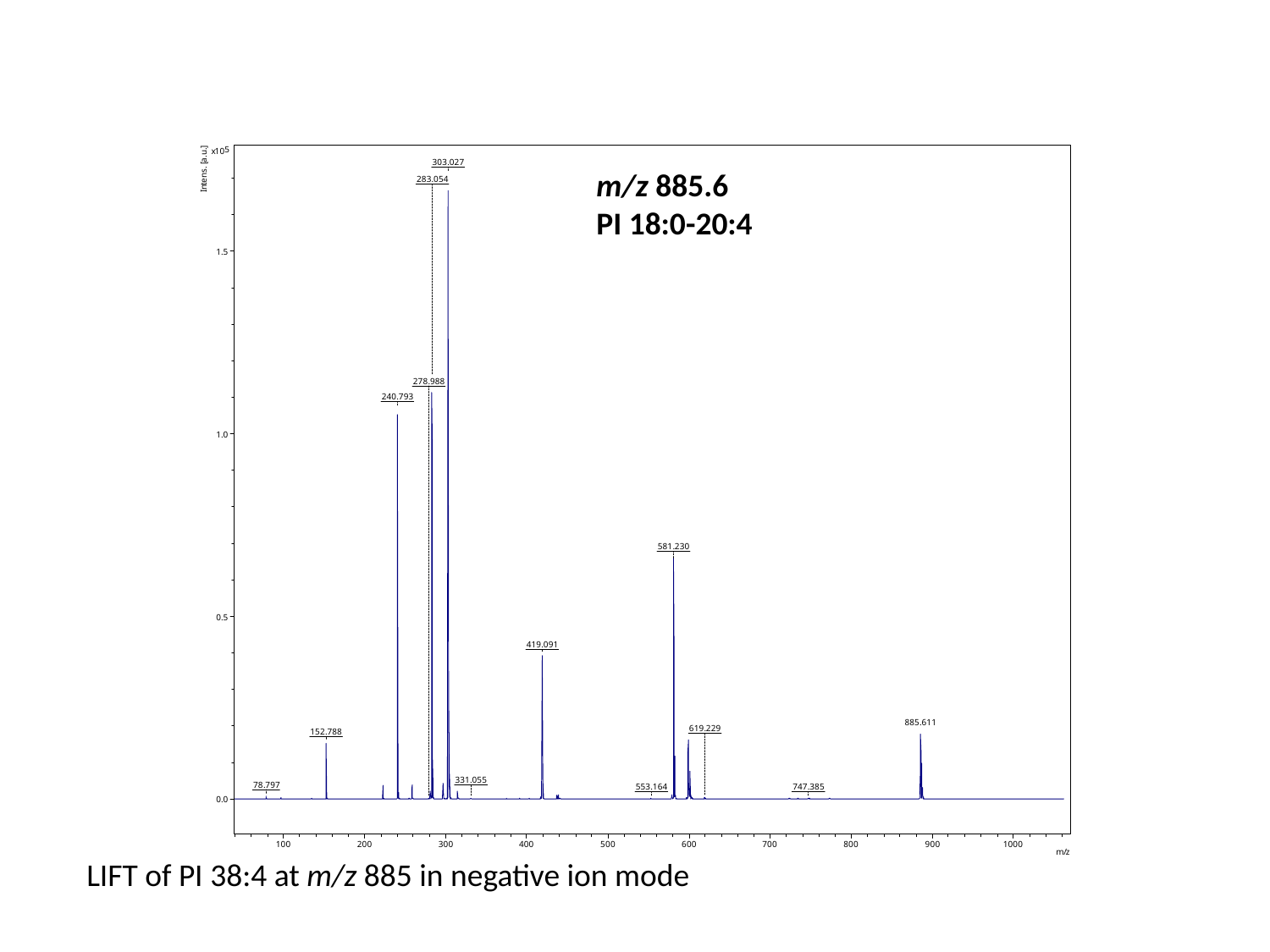

m/z 885.6
PI 18:0-20:4
LIFT of PI 38:4 at m/z 885 in negative ion mode

## Slide 3
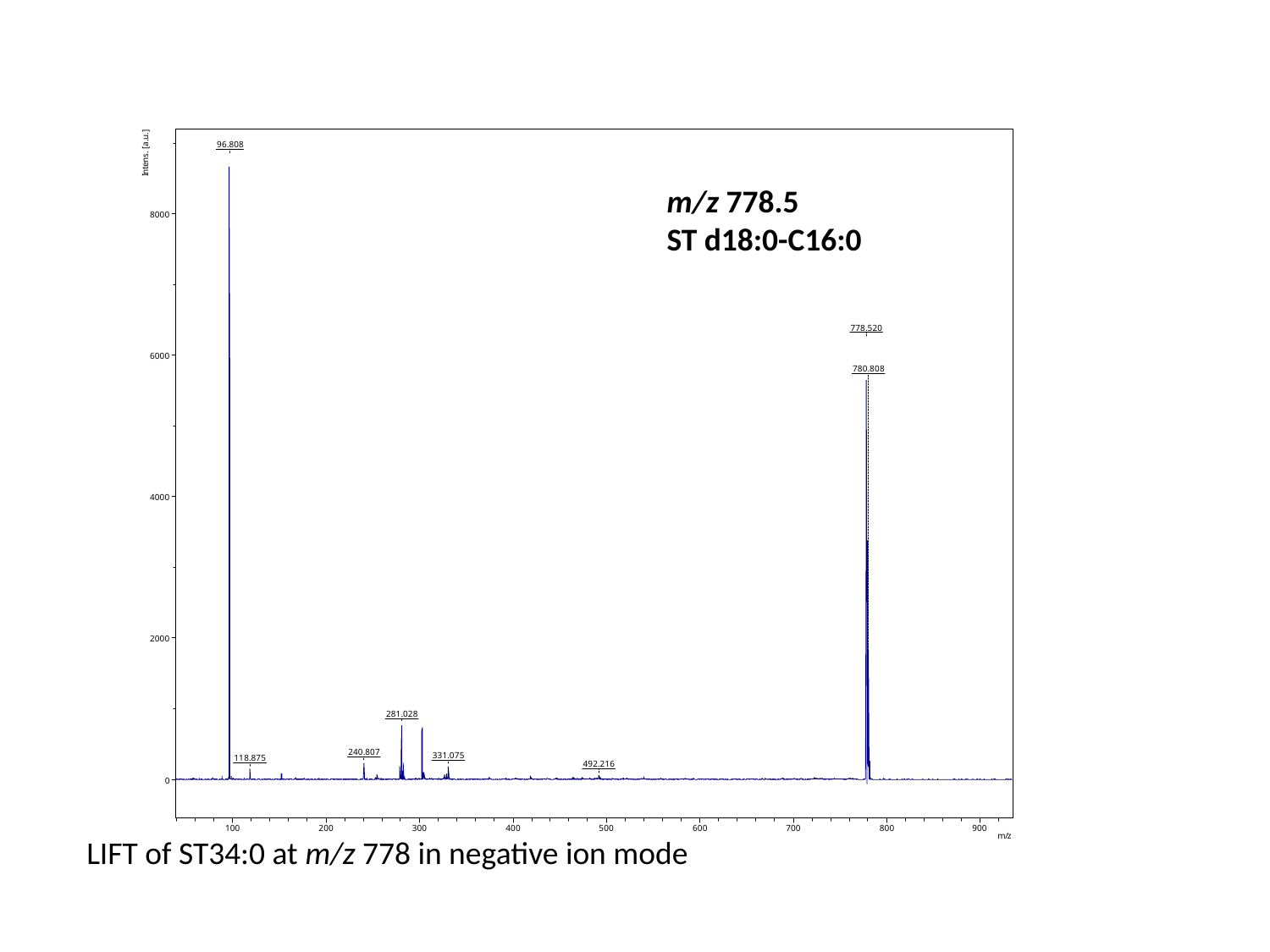

m/z 778.5
ST d18:0-C16:0
LIFT of ST34:0 at m/z 778 in negative ion mode

## Slide 4
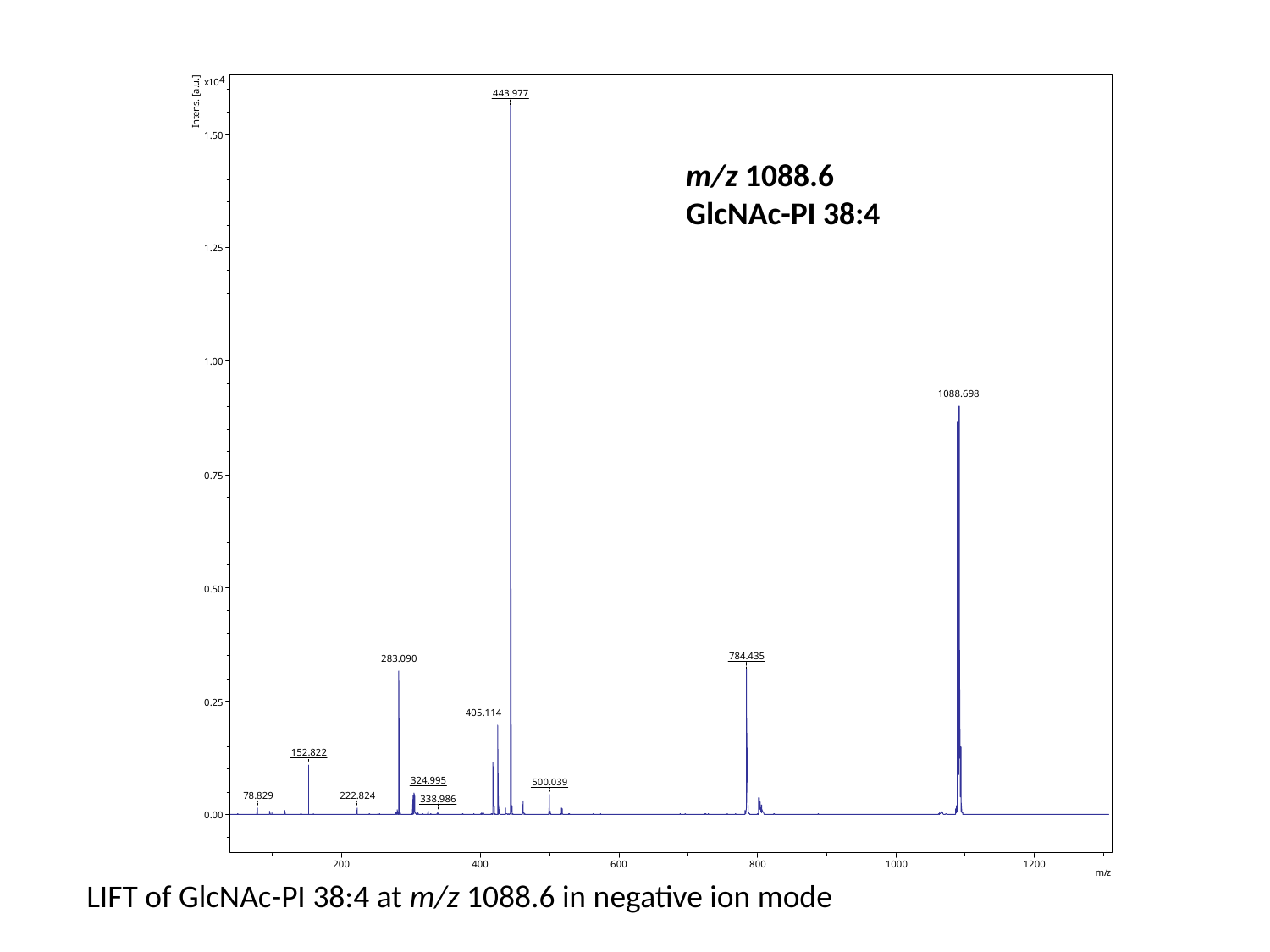

m/z 1088.6
GlcNAc-PI 38:4
LIFT of GlcNAc-PI 38:4 at m/z 1088.6 in negative ion mode

## Slide 5
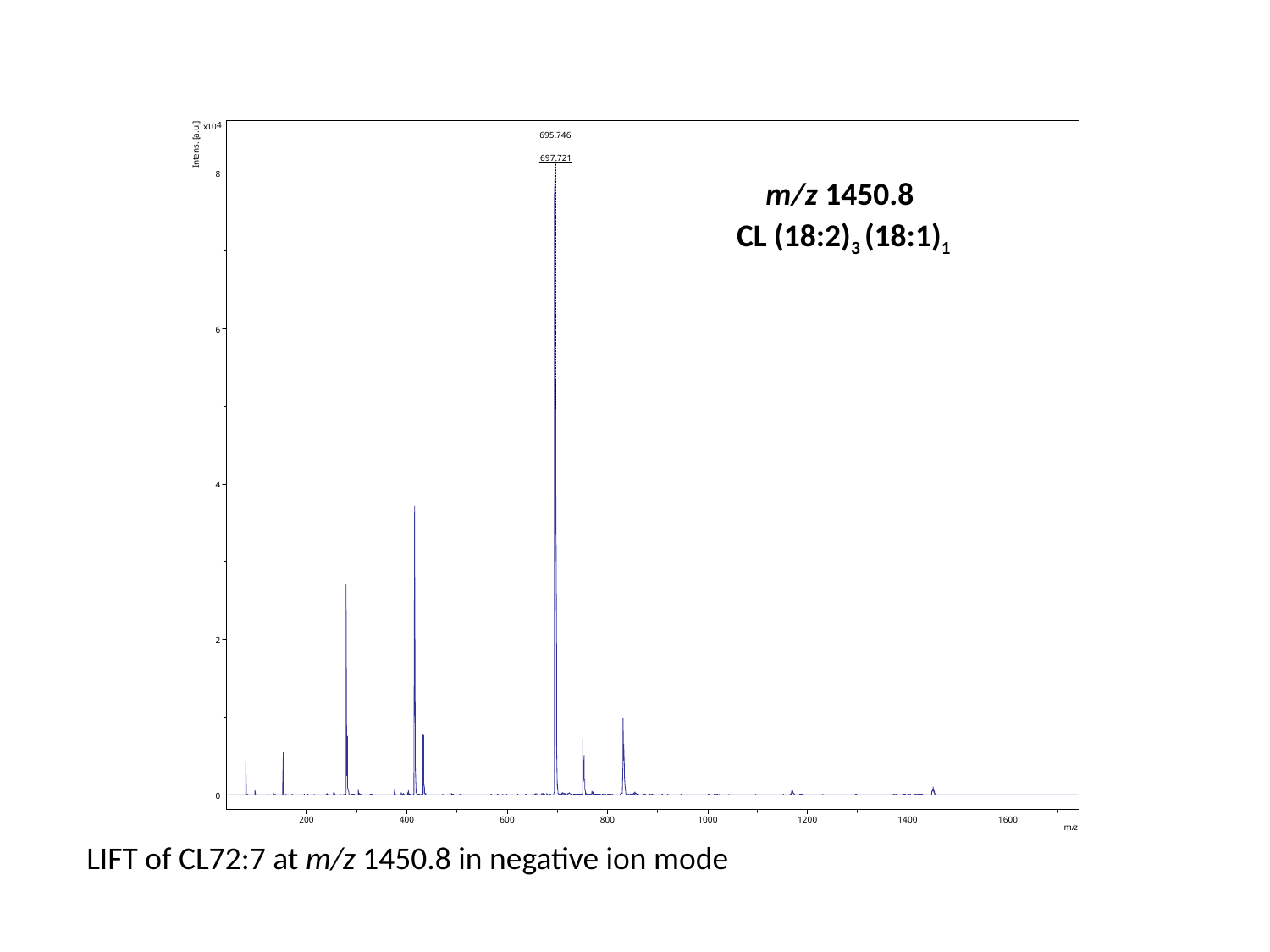

m/z 1450.8
CL (18:2)3 (18:1)1
LIFT of CL72:7 at m/z 1450.8 in negative ion mode
